# Combinations of scleroderma hallmark autoantibodies associate with distinct clinical phenotypes

**DOI:** 10.1038/s41598-022-15062-4

**Published:** 2022-07-02

**Authors:** Kristina E. N. Clark, Corrado Campochiaro, Lauren V. Host, Alper Sari, Jennifer Harvey, Christopher P. Denton, Voon H. Ong

**Affiliations:** 1 Centre for Rheumatology and Connective Tissue Diseases, Division of Medicine, University College London, London, UK; 2 Unit of Immunology, Rheumatology, Allergy and Rare Diseases, IRCCS San Raffaele Hospital, Vita-Salute San Raffaele University, Milan, Italy; 3 Department of Internal Medicine, Faculty of Medicine, Hacettepe University, Ankara, Turkey

**Keywords:** Autoimmunity, Rheumatic diseases

## Abstract

Systemic sclerosis (SSc) is characterized by the presence of SSc-specific or SSc-associated antibodies (SSc-Abs): anti-topoisomerase I (ATA), anti-centromere (ACA), anti-RNA polymerase III (ARA), anti-U3RNP (U3RNP), anti-U1RNP (U1RNP), anti-PmScl (PmScl), anti-Ku (Ku) and anti-Th/To (Th/To), each being associated with specific clinical features and prognosis. The detection of more than one SSc-Abs in SSc patients is rare and only few data about these patients’ clinical phenotype is available. The aim of our study was to evaluate the frequency and the disease’s features associated with the presence of > 1 SSc-Abs positivity in a large cohort of SSc patients. The autoantibody profiles of 2799 SSc patients from February 2001 to June 2017 were retrospectively reviewed. Patients with > 1 SSc-Abs were identified. Clinical features were collected and compared to a large historical cohort of SSc patients with single SSc-Ab positivity. SSc patients were excluded if previously treated with rituximab, intravenous immunoglobulins or stem cell transplantation. Non-parametric tests were used for statistical analysis. Nearly 5% of SSc patients from our cohort had ≥ 2 autoantibody positivity, and 2.3% (n = 72) had ≥ 2 SSc-Abs positivity. Th e most common combination was U1RNP and ATA (35%). These patients were younger than patients with single autoantibody positivity and showed more commonly a diffuse cutaneous SSc form. They also had higher rates of overlap features compared to ATA patients. Other combinations included U1RNP and ACA (13%), ATA and ACA (7%) and U1RNP and PmScl (5%). In our study we observed that, while infrequently, SSc patients can present with a combination of two SSc-Abs and that the double positivity can influence their clinical phenotype compared to patients with single SSc-Ab positivity. The importance of re-testing SSc-Abs in patients with changing clinical phenotypes was also highlighted, as this may confer a differing risk stratification.

## Introduction

The serological hallmark of systemic sclerosis (SSc) is the presence of specific autoantibodies and up to 95% of patients with SSc will have at least one autoantibody^1^. Certain autoantibodies are specific to SSc and are included in the 2013 ACR/EULAR (American College Rheumatology/European League against Rheumatism) SSc classification criteria^2^. These include anti-topoisomerase antibody (ATA), anti-centromere antibody (ACA), and anti RNA-Polymerase III antibody (ARA) as well as anti-U3-RNP (U3RNP). These four antibodies account for up to 80% of the anti-nuclear antibodies (ANA) detected in SSc. The other specific autoantibodies identified less frequently include anti-Th/To (Th/To), anti-Ku (Ku), anti-PmScl (PmScl) and anti-U1-RNP (U1RNP). Autoantibodies are associated with differing phenotypes, and are important for both stratifying patients by their risk for developing organ complications, as well as overall survival^3–5^.

ACA and ATA are the commonest autoantibodies in the SSc population. ACA is found in 20–38% of patients, and most commonly associated with limited SSc (lcSSc), where it is found in over 50% of patients^3,6^. ATA is found in 15–42% of patients, particularly diffuse SSc (dcSSc), and confers an increased risk for pulmonary fibrosis (up to 70% of patients). ARA is another dcSSc predominant autoantibody in 5–31% patients, and is associated with renal crisis in 45% of patients^3^.

Up to 20% of patients with SSc will have an overlap syndrome^7,8^, the commonest being myositis, rheumatoid arthritis and Sjögren syndrome. However this is based on clinical features rather than serology. There is a relatively frequent co-existence of non-SSc specific autoantibodies, most commonly anti-Ro (in up to 56% of patients with ACA)^9^.

Traditionally the SSc specific autoantibodies were thought to be mutually exclusive^6,9–12^, but there is a small body of literature on their coexistence. The most commonly reported SSc specific dual positivity is the co-existence of ATA and ACA (0.05–0.6% of SSc patients^13,14^). The clinical phenotype of this combination of autoantibodies seems to mirror the ATA population with 60% of patients being dcSSc, and having clinical and visceral symptoms in keeping with ATA single positivity.

Previous works have not focused on clinical phenotypes of other double SSc specific autoantibody positivity. Therefore, the aim of this study was to determine the frequency and clinical phenotypes of patients with the co-existence of more than one SSc specific autoantibodies from our tertiary referral centre for connective tissue diseases.

## Methods

The autoantibody (Ab) profiles of 2799 SSc patients from February 2001 to June 2017 were retrospectively reviewed. All patients met the ACR/EULAR 2013 classification criteria for SSc. Patients with > 1 positivity at any time for the following SSc-specific or SSc-associated antibodies were selected: ACA, ATA, U3RNP, Ku, PmScl, U1RNP, Th/To, ARA. Clinical features were collected for these patients, in particular: age, sex, presence of overlap features, SSc subtype, inflammatory arthritis, lung fibrosis on computer tomography (CT), pulmonary arterial hypertension (PAH), scleroderma renal crisis (SRC), cardiac involvement and skeletal myositis. SSc subtype was classified as either lcSSc or dcSSc. Lung fibrosis was defined as the presence of interstitial lung disease (ILD) at CT scan. PAH was diagnosed after right heart catheter in all patients. Cardiac involvement was defined as the presence of diagnostic cardiac biopsy, cardiac magnetic resonance features or the necessity of introduction anti-arrhythmic therapy not explained by other medical conditions. For comparison we included an historical SSc cohort of patients with single positivity for each of the autoantibodies initially selected and followed-up at the same referral centre.

Antibody testing was performed in the hospital immunology laboratory. Antinuclear antibody (ANA) positivity was confirmed by indirect immunofluorescence (IIF) on Hep-2 cells. Positivity for ACA, anti-U3-RNP, and anti-Th/T0 was based on their specific staining pattern. The staining pattern used to define anti-U3-RNP was described by Tormey et al.^15^. The pattern consisted of three elements – clumpy nucleolar staining with staining of the coilin bodies in interphase cells and classic fibrillar or lacy staining in metaphase cells. The staining pattern used to describe anti-Th/T0 was homogeneous staining of the nucleoli with fine speckled staining of the nucleoli with fine speckled staining of the nucleoplasm^16^. The immunology laboratory performed an internal validation of the method, by correlating the pattern observed on indirect immunofluorescence (IIF) and immunoprecipitation (IP) testing, although the results have not been published. Positivity for ARA was confirmed on ELISA. ATA, anti-Pm/Scl, anti-U1-RNP, anti-Ku antibody positivity was tested using counterimmunoelectrophoresis.

Among > 1 antibody positive patients we identified a separate cohort who had a sequential accumulation of antibodies (referred to as “gain antibody cohort”), rather than 2 antibodies from initial testing. For these patients the time interval between additional antibody acquirement was recorded, as well as any change in clinical phenotype. Among the gain antibody patients we then additionally identified patients who had concomitantly lost and acquired a new antibody (“switch antibody cohort”).

To avoid drug-related autoantibody misinterpretations, we excluded patients who had been treated with rituximab, intravenous immunoglobulins (IvIg) or stem cell transplantation prior to the immunology tests.

Statistical analysis were performed using SPSS versions 22. Continuous variables are expressed as mean ± standard deviations (SD). Categorical variables are expressed as number (%). Two-sided Fisher’s exact test and Mann–Whitney U test were used to perform comparisons. A *p* value < 0.05 was considered statistically significant.

This study was conducted in compliance with the Declaration of Helsinki. Informed consent was obtained from patients routinely reviewed in our centre which has been approved by the London-Hampstead and the London-Fulham Research Ethics Committees.

### Ethics approval and consent to participate

All patients gave consent for their anonymous data to be used.

## Results

### Study cohort

We initially identified 122 patients (4.3% of our cohort) with greater than one autoantibody where at least one autoantibody was SSc-specific or SSc-associated. We then focused on patients with SSc specific autoantibodies, and identified 72 patients (2.6% of our cohort) with at least two SSc specific autoAb. Full clinical data were available for 63 patients. In particular we found 60 patients (2.1%) with double Ab positivity and 3 patients with triple Ab positivity (0.1%). The antibody most frequently associated with a double positivity was U1RNP (43 patients, 72%), followed by ATA (21 patients 35%) and ACA (19 patients, 32%) (See Table 1). Anti Th/T0 was not found in combination with any of the other antibodies. Interestingly all patients with three autoantibodies present were positive for anti-U1RNP antibody. In our cohort the most frequent combination was U1RNP and ATA (21 patients, 35%) followed by U1RNP and ACA (8 patients, 13%) and U1RNP and U3RNP (6 patients, 10%).

**Table 1:** Frequency of antibody combinations.

Antibody combination	Number of patients (%)
*U1RNP*	43 (72%)
ATA	21 (35%)
ACA	8 (13%)
U3RNP	6 (10%)
ARA	4 (7%)
PmScl	3 (5%)
Ku	1 (2%)
ACA and ATA	1 (2%)
U3RNP and Ku	1 (2%)
ATA and U3RNP	1 (2%)
*ATA*	29 (48%)
ACA	4 (7%)
Ku	3 (5%)
ARA	1 (2%)
*ACA*	19 (32%)
PmScl	4 (7%)
Ku	1 (2%)
U3RNP	2 (3%)
*PmScl*	9 (15%)
Ku	2 (3%)

### Clinical features

We found a total of 13 possible Ab combinations (Table 1). For each combination, the clinical features of patients were compared to a cohort of 978 monoAb patients that included 382 ACA patients, 268 ATA patients, 143 ARA patients, 50 U3RNP patients, 76 U1RNP patients, 5 Ku patients and 54 PmScl patients.

#### U1RNP and ATA

This was the largest cohort with 21 patients (35%). When comparing U1RNP and ATA positive patients we found they were significantly younger (51.38 ± 11.56 years) than both U1RNP (58.64 ± 13.10 years, *p* = 0.050) and ATA (62.03 ± 15.04 years, *p* = 0.002) patients and they were more commonly of diffuse cutaneous subset (76% vs 21%, *p* = 0.001 and 75% vs 52%, *p* = 0.041 respectively) (Table 2). We also found that compared to ATA patients they had significantly more frequently overlap features (43% vs 15%, *p* = 0.004) including inflammatory arthritis (29% vs 10%, *p* = 0.025) and myositis (19% vs 4%, *p* = 0.013) (Table 3).

**Table 2:** Table comparing demographics of double positivity SSc specific autoantibody group and cohort of patients with individual autoantibody.

Double antibody group	Comparator group	Demographics							
		Age (years)		Sex (male)		Overlap		SSc diffuse	
		N	*P*	N	*p*	n	*p*	n	*p*
U1 RNP and ATA (n = 21)		51.38 ± 11.56		3 (14%)		9 (43%)		16 (76%)	
	U1RNP	58.64 ± 13.10	**0.05**	15 (20%)	0.755	40 (53%)	0.464	16 (21%)	**0.001**
	ATA	62.03 ± 15.04	**0.002**	50 (19%)	0.775	41 (15%)	**0.004**	140 (52%)	**0.041**
U1RNP and ACA (n = 8)		57.88 ± 10.87		1 (12%)		3 (37%)		2 (25%)	
	U1RNP	58.64 ± 13.10	0.873	15 (20%)	1	40 (53%)	0.473	16 (21%)	0.678
	ACA	68.75 ± 12.61	**0.015**	34 (9%)	0.532	59 (15%)	0.119	10 (3%)	**0.022**
U1RNP and ARA (n = 4)		58.0 ± 8.76		1 (25%)		0		3 (75%)	
	U1RNP	58.64 ± 13.10	0.923	15 (20%)	1	40 (53%)	**0.05**	16 (21%)	**0.04**
	ARA	64.46 ± 11.92	0.161	29 (20%)	1	12 (8%)	1	126 (88%)	0.41
U1 RNP and PmScl (n = 3)		50.67 ± 24.44		1 (33%)		3 (100%)		0	
	U1RNP	58.64 ± 13.10	0.319	15 (20%)	0.498	40 (53%)	0.248	16 (21%)	1
	PmScl	62.20 ± 14.19	0.191	11 (20%)	0.515	27 (50%)	0.239	20 (37%)	0.545
ATA and ACA (n = 4)		62.25 ± 10.21		0		1 (25%)		1 (25%)	
	ATA	62.03 ± 15.04	0.67	50 (19%)	1	41 (15%)	0.496	140 (52%)	1
	ACA	68.75 ± 12.61	0.581	34 (9%)	1	59 (15%)	0.493	10 (3%)	0.11
ACA and PmScl (n = 4)		69.25 ± 20.22		0		2 (50%)		1 (25%)	
	ACA	68.75 ± 12.61	1	34 (9%)	1	59 (15%)	0.119	10 (3%)	0.11
	PmScl	62.20 ± 14.19	0.355	11 (20%)	1	27 (50%)	1	20 (37%)	1
ACA and U3RNP (n = 2)		51.50 ± 2.12		0		1 (50%)		0	
	ACA	68.75 ± 12.61	**0.045**	34 (9%)	1	59 (15%)	0.288	10 (3%)	1
	U3RNP	59.70 ± 14.89	0.466	19 (38%)	0.527	11 (22%)	0.419	29 (58%)	0.191
PmScl and Ku (n = 2)		49.0 ± 1.41		0		2 (100%)		0	
	PmScl	62.20 ± 14.19	0.198	11 (20%)	1	27 (50%)	0.492	20 (37%)	0.532
	Ku	70.2 ± 24.49	0.3	1 (20%)	1	2 (40%)	0.429	1 (20%)	1
U1RNP and U3 RNP (n = 6)		55.50 ± 8.26		0		2 (33%)		3 (50%)	
	U1RNP	58.64 ± 13.10	0.566	15 (20%)	0.586	40 (53%)	0.421	16 (21%)	0.134
	U3RNP	59.70 ± 14.89	0.503	19 (38%)	0.086	11 (22%)	0.619	29 (58%)	1
ATA and Ku (n = 3)		56.0 ± 2.64		0		1 (33%)		2 (66%)	
	ATA	62.03 ± 15.04	0.489	50 (19%)	1	41 (15%)	0.403	140 (52%)	1
	Ku	70.2 ± 24.49	0.489	1 (20%)	1	2 (40%)	1	1 (20%)	0.464

Significant *p* values (< 0.05) are highlighted in bold.

**Table 3:** Comparison between clinical features of double positivity SSc specific autoantibody group, and cohort of patients with single autoantibody positivity.

Double antibody group	Comparator group	Clinical features											
		Arthritis		ILD		PAH		SRC		Cardiac		Myositis	
		n	*p*	n	*p*	n	*p*	n	*p*	n	*p*	n	*p*
U1 RNP and ATA		6 (29%)		15 (68%)		1 (9%)		0		0		4 (19%)	
	U1RNP	14 (18%)	1	38 (50%)	0.136	12 (16%)	0.287	1 (1%)	1	2 (3%)	1	12 (16%)	0.744
	ATA	28 (10%)	**0.025**	213 (79%)	0.382	12 (4%)	1	17 (6%)	0.622	20 (7%)	0.378	10 (4%)	**0.013**
U1RNP and ACA		2 (25%)		2 (25%)		4 (50%)		0		0		3 (37%)	
	U1RNP	14 (18%)	1	38 (50%)	0.267	12 (16%)	**0.039**	1 (1%)	1	2 (3%)	1	12 (16%)	0.148
	ACA	23 (6%)	0.087	43 (11%)	0.233	56 (15%)	**0.022**	6 (2%)	1	10 (3%)	1	3 (1%)	**0.001**
U1RNP and ARA		0		2 (50%)		0		1 (25%)		1 (25%)		0	
	U1RNP	14 (18%)	0.311	38 (50%)	1	12 (16%)	1	1 (1%)	0.098	2 (3%)	0.144	12 (16%)	1
	ARA	4 (3%)	1	64 (45%)	1	15 (10%)	1	34 (24%)	1	4 (3%)	0.131	5 (3%)	1
U1 RNP and PmScl		1 (33%)		2 (66%)		0		0		0		3 (100%)	
	U1RNP	14 (18%)	1	38 (50%)	1	12 (16%)	1	1 (1%)	1	2 (3%)	1	12 (16%)	**0.006**
	PmScl	7 (13%)	1	27 (50%)	1	3 (6%)	1	3 (6%)	1	1 (2%)	1	21 (39%)	0.069
ATA and ACA		0		3 (75%)		0		0		0		1 (25%)	
	ATA	28 (10%)	1	213 (79%)	1	12 (4%)	1	17 (6%)	1	20 (7%)	1	10 (4%)	0.153
	ACA	23 (6%)	1	43 (11%)	**0.006**	56 (15%)	1	6 (2%)	1	10 (3%)	1	3 (1%)	**0.041**
ACA and PmScl		1 (25%)		1 (25%)		0		0		0		1 (25%)	
	ACA	23 (6%)	0.227	43 (11%)	0.385	56 (15%)	1	6 (2%)	1	10 (3%)	1	3 (1%)	**0.04**
	PmScl	7 (13%)	0.457	27 (50%)	0.612	3 (6%)	1	3 (6%)	1	1 (2%)	1	21 (39%)	1
ACA and U3RNP		0		1 (50%)		0		0		0		0	
	ACA	23 (6%)	1	43 (11%)	0.216	56 (15%)	1	6 (2%)	1	10 (3%)	1	3 (1%)	1
	U3RNP	3 (6%)	1	12 (24%)	0.456	13 (26%)	1	5 (10%)	1	6 (12%)	1	9 (18%)	1
PmScl and Ku													
	PmScl	7 (13%)	0.268	27 (50%)	1	3 (6%)	1	3 (6%)	1	1 (2%)	1	21 (39%)	1
	Ku	1 (20%)	1	2 (40%)	1	0	1	0	1	1 (20%)	1	2 (40%)	1
U1RNP and U3 RNP													
	U1RNP	14 (18%)	1	38 (50%)	0.675	12 (16%)	0.271	1 (1%)	0.142	2 (3%)	0.206	12 (16%)	0.585
	U3RNP	3 (6%)	0.084	12 (24%)	0.643	13 (26%)	0.654	5 (10%)	0.511	6 (12%)	0.569	9 (18%)	0.575
ATA and Ku													
	ATA	28 (10%)	0.289	213 (79%)	1	12 (4%)	0.138	17 (6%)	1	20 (7%)	1	10 (4%)	1
	Ku	1 (20%)	1	2 (40%)	0.196	0	0.375	0	1	1 (20%)	1	2 (40%)	0.464

Significant *p* values (< 0.05) are highlighted in bold. ILD (interstitial lung disease), PAH (pulmonary arterial hypertension), SRC (scleroderma renal crisis).

#### U1RNP and ACA

This was the second most frequent combination of Abs, with 8 patients (13%). They had a significantly higher prevalence of PAH (50%) compared to both U1RNP (16%, *p* = 0.039) and ACA (15%, *p* = 0.022) patients. Compared to ACA patients they were also significantly younger (57.88 ± 10.87 vs 68.75 ± 12.61, *p* = 0.015) and more frequently affected by myositis (37% vs 1%, *p* = 0.001).

#### U1RNP and ARA

This group included 4 patients (7%). Compared to U1RNP patients, this cohort had no features of overlap disease (0 vs 53%, *p* = 0.050) and were more frequently of diffuse cutaneous subtype (75% vs 21%, *p* = 0.040). Frequency of clinical features were similar compared to patients with each individual autoantibody.

#### U1RNP and PmScl

This group included 3 patients. The only significant clinical feature was a higher prevalence of skeletal myositis in the double positive patients compared to U1RNP patients (100% vs 16%, *p* = 0.006).

#### ATA and ACA

This interesting group included 4 patients (7%). When comparing ATA and ACA patients to ACA patients and ATA patients we found these patients behaved more similarly to ATA than ACA as they had a significantly higher prevalence of lung fibrosis compared to ACA (75% vs 11%, *p* = 0.006) and of skeletal myositis (25% vs 1%, *p* = 0.041).

#### ACA and PmScl

This group included 4 patients. The only significant clinical feature was a higher prevalence of myositis compared to ACA patients (25% vs 1%, *p* = 0.04).

#### ACA and U3RNP

This group included only two patients. The only significant clinical feature was a younger age of disease onset compared to ACA patients (51.50 ± 2.12 vs 68.75 ± 12.61, *p* = 0.045).

#### Other combinations

No significant clinical features were associated with the other Ab combinations.

### Gain antibody

32 patients out of 2799 (1.14%) were identified as gaining at least 1 autoantibody during the study period. Of these, 15 patients initially had one SSc specific autoantibody, and gained a second SSc specific autoantibody. One patient gained 2 SSc specific autoantibodies, and 1 patient started with 2 SSc specific Abs and gained a third. The rest gained a non-SSc specific autoantibody and were not included in the subsequent analysis (Table 4).

**Table 4:** Table to show sequential gain in autoantibodies, and those associated with clinical change.

First Ab	Second Ab	Third Ab	Significant change in clinical phenotype
ATA	U1RNP		Panniculitis
ACA	U1RNP		No
U1RNP	ARA		Developed dcSSc from lcSSc
ACA	PmScl		No
U3RNP	ACA		No
U1RNP	ARA		No
U1RNP	U3RNP		Myositis
U3RNP	ACA		No
ATA	U1RNP	ACA	With U1RNP developed ILD, and dcSSc. With ACA- new onset severe DUs
PmScl	ACA		No
ATA	U1RNP		ILD
ATA	Ku		No
Ku	ATA		PAH
ATA	ACA		No
ATA	U1RNP		No
U3RNP, U1RNP	Ku		Myositis, cardiac
ATA	Ku		No

DUs, digital ulcers; ILD, interstitial lung disease; dcSSc, diffuse cutaneous systemic sclerosis; lcSSc, limited cutaneous systemic sclerosis.

The most common initial autoantibody was ATA (7 patients 38.9%), followed by U1RNP (4 patients 12.5%). The median time interval between first autoantibody identification and second autoantibody identification was 38 months (range 5–183 months). The most common second Abs were ACA and U1RNP (5 respectively, 29.4%).

The gain of a new SSc specific autoantibody was associated with a clinical modification in 7 patients (38.9%) (Table 4). 50% of the patients who started with ATA and gained U1RNP developed ILD (interstitial lung disease). In 2 patients the gain in autoantibody was associated with a switch from lcSSc to dcSSc.

### Switch antibody

A total of 4 patients out of 72 double antibody patients (5%) simultaneously gained and lost an antibody. We have called this group of patients “switch autoantibody” (see Table 5).

**Table 5:** Cohort of patients who initially presented with one autoantibody, and during their condition lost their initial SSc specific Ab, and gained a different one (“switch patients”).

Antibody lost	Antibody gained	Change in clinical phenotype
ARA	PmScl	No
U3RNP	U1RNP	ILD
ATA	ARA	ILD progression
U3RNP	Ku	Myositis

The clinical change is also documented.

Median time interval was 90.5 (77.25 – 98.5) months between change in autoantibody. In 3 cases (75%) there was a significant clinical modification of disease’ features upon Ab profile modification, in particular one patient developed ILD, one patient had a notable ILD progression, and one patient developed myositis. None of these patients had any medication which could have altered their autoantibody profile.

## Discussion

We present our tertiary centre experience of the clinical phenotype of double SSc specific antibody positivity, either at initial testing, or sequential addition of autoantibodies with subsequent testing. We focused only on SSc specific autoantibodies, as they traditionally have been described as being mutually exclusive. There has been previous literature discussing double autoantibody with anti-Ro, or anti-La, but these can be non-specific findings, and are more frequently found in combination than the SSc-specific or SSc-associated autoantibodies. Indeed, in a previous study it was found that anti-Ro and/or anti-La positivity were only marginally associated with sicca syndrome and that the positivity for anti-Ro60 was found in strong association with pulmonary fibrosis, but this finding was deemed secondary to the high co-occurrence of anti-Ro60 with ATA^17^.

The prevalence of double autoantibody in the literature in all SSc patients ranges from 0.6–5%, with one study reporting up to 35%^18,19^. The larger proportion of SSc double antibody positivity was though not limited to SSc specific autoantibodies, and therefore is likely to account the disproportionate high frequency. Our cohort was comparable with a prevalence of 4.3% having double autoantibody positivity, and 2.3% of our cohort having at least two SSc specific autoantibodies.

Interestingly, we observed not only that the presence of two SSc-specific or SSc-associated autoAbs can influence patients’ clinical phenotype but that each autoAb could have a different clinical expression according to the combination observed.

In terms of predominating autoantibody, we found that in the U1RNP and ATA combination, clinical features were more consistent with U1RNP. This was mirrored in the U1RNP and ACA combination, although they had a higher risk of PAH compared to U1RNP on its own suggesting a synergistic effect on the two Abs on the development of PAH. In the U1RNP and PmScl combination, clinical features consistent with PmScl dominated, as did the clinical features of PmScl in the ACA and PmScl combination. In keeping with previous literature, the clinical features of ATA dominated in the double positivity patients of ATA and ACA^14^.

The combination of ACA and ATA has been previously described by numerous cohorts, and ranges between 0.5–5%^13,14,18,19^. Unlike previous studies, the majority of our patients had lcSSc, however they had comparable clinical phenotypes to the single positive ATA patients. Traditionally patients with ATA have a higher frequency of dcSSc. Other studies have reported increased rates of calcinosis and vascular complications compared to ACA or ATA alone^20^, however our clinical records did not allow for interpretation of this.

The most frequent occurring double autoantibody group was U1-RNP and ATA, and within our whole population, made up 0.8% of the patients included in the analysis. These patients were typically younger with higher rate of dcSSc disease. Their clinical phenotype was more comparable to U1RNP than ATA. Consistent with a previous large study not limited to SSc patients, compared to ATA, this combination was more likely to be observed in patients with overlap disease, myositis and arthritis^21^.

ARA and U1 RNP has also previously been reported. In one small case series, 2 patients had SLE, and only 1 had SSc overlap. Unlike this study, none of our patients had overlap features, and the majority of patients had dcSSc^22^, however we only looked at a cohort of patients with established SSc.

Previous studies have focused more on the differing techniques used to identify > 1 autoantibody in the serum of individual patients (immunoblot or ELISA), rather than the clinical phenotypes of the patients with these autoantibody profiles^9,18,23^. All our autoantibody testing was derived from the same laboratory, and utilised the same techniques throughout the study period. This therefore would not explain the sequential gain in autoantibodies, as techniques were not changed in between sample analysis.

### Discussion on gain SSc specific

It is appreciated that in SSc, autoantibodies remain present throughout the course of the disease, with little variability in titres, except possibly in very mild cases^24–26^. The prospective gain of additional SSc specific autoantibodies has very rarely been reported in the literature. In our cohort we identified 15 patients who gained an additional SSc specific autoantibody during the course of their disease, and in 7 of these patients, it was associated with a clinical meaningful change in their disease.

Although it is not our routine practice, these results highlight the potential value of repeat autoantibody testing, especially in the presence of a change in clinical manifestations. Given that this is not routinely done, we do not know the time delay between the gain in the additional SSc specific autoantibody. The number of gain autoantibodies may also be vastly underestimated as many patients may not have had repeat autoantibody testing within the study period. The knowledge of any additional autoantibody is clinically relevant in prognosticating patients for potential organ involvement. Further work is needed to decide whether regular testing of autoantibodies would be of merit.

### Discussion on switch autoantibodies

There is very little in the literature of the clinical impact of seroconversion of autoantibodies in SSc. In our cohort, 4 patients out of the 72 identified as having more than one SSc specific autoantibody had initially presented with one autoantibody, and switched to another during the course of this disease. In 3 of these patients, a modification of their clinical phenotype was identified. There has been only one case report of a patient converting from anti Th/To to ACA^27^, although neither of these autoantibodies were present in our seroconversion cohort. The seroconversion also predated any change in clinical phenotype. We do not know if this would be the case with our cohort, as autoantibodies are not routinely tested, and a change in the clinical picture may prompt a repeat testing of the autoantibodies.

### Limitations

Given the rarity of having two SSc specific autoantibodies, some of the cohorts used in our analysis are very small, and therefore conclusions may not be fully accurate. Moreover, as re-testing of autoantibodies was not routinely performed in our cohort, the real prevalence of double SSc-specific or SSc-associated Abs might be underestimated.

This is a retrospective analysis based on immunology results, and available clinical information, which may impact the reliability of all clinically available information.

## Conclusion

We present our cohort of patients with at least two SSc specific autoantibodies. Our work highlights that the traditional teaching that these show mutual exclusivity is not wholly accurate, and a small but significant proportion of patients do have more than one SSc specific autoantibody. This double antibody positivity in the majority of patients does confer differing clinical phenotype, either in terms of demographics, or clinical manifestations, compared to those patients with only one of the autoantibodies present. This work also highlights the importance of re-testing immunological profiles with change in clinical phenotypes, as patients may have gained a new autoantibody, and thus their risk stratification profile may also have changed. Prospective studies are needed to identify the patients who will benefit from repeated.

## Data Availability

The datasets used and/or analysed during the current study are available from the corresponding author on reasonable request.
